# Frk positively regulates innate antiviral immunity by phosphorylating TBK1

**DOI:** 10.3389/fmicb.2025.1525648

**Published:** 2025-02-12

**Authors:** Xiaomei Zhang, Ying You, Tingrong Xiong, Xiaokai Zhang, Haibo Wang, Jinxia Geng, Miao Wang, Yanyan Xu, Shanshan Gao, Xiaoyan Wu, Yue Zheng, Xianhua Wen, Haoyu Yang, Yu Wang, Xiaohua Wen, Congcong Zhao

**Affiliations:** ^1^Department of Medical Engineering, Xinqiao Hospital, Third Military Medical University (Army Medical University), Chongqing, China; ^2^Clinical Medical Research Center, Southwest Hospital, Third Military Medical University, Chongqing, China; ^3^Department of Microbiology and Biochemical Pharmacy, National Engineering Research Center of Immunological Products, College of Pharmacy, Third Military Medical University, Chongqing, China; ^4^Chongqing Key Laboratory of Natural Product Synthesis and Drug Research, School of Pharmaceutical Sciences, Chongqing University, Chongqing, China; ^5^Department of Basic Courses, Non Commissioned Officer School, Third Military Medical University, Shijiazhuang, China; ^6^Department of Health Medicine, The 980th Hospital of People’s Liberation Army Joint Logistics Support Forces, Shijiazhuang, China

**Keywords:** macrophages, Frk, TBK1, phosphorylation, IFN-I

## Abstract

Type I interferons (IFN-I) are crucial for the initial defense against viral infections. TBK1 serves as a key regulator in the production of IFN-I, with its phosphorylation being essential for the regulation of its activity. However, the regulatory mechanisms governing its activation remain incompletely elucidated. In this study, we validated the function of Fyn-related kinase (Frk) in the antiviral innate immune response and identified the direct target molecule of Frk in the IFN-β signaling pathway. Furthermore, we elucidated the mechanism by which Frk phosphorylates TBK1 during infection and the role of Frk in IFN-β production. We discovered that Frk enhances the activation of the IFN-I production pathway by targeting TBK1. Mechanistically, Frk promotes the K63 ubiquitination of TBK1 and subsequent activation of the transcription factor IRF3 by phosphorylating TBK1 at tyrosine residues 174 and 179, thereby enhancing the production of IFN-β in macrophages. Employing both *in vivo* and *in vitro* viral infection assays, we demonstrated that IFN-β mediated by Frk inhibits the replication of VSV or HSV-1 and alleviates lung lesions. Our findings indicate that Frk functions as a key regulator of TBK1 to strengthen antiviral immunity and represents a promising target for the development of antiviral drugs.

## Introduction

Viruses pose substantial challenges to the realm of international public health, with numerous aspects of their prevention and treatment that remain effectively unaddressed (The Lancet, 2020; Holshue et al., 2020; Rothe et al., 2020). The innate antiviral immune reactions, particularly the signaling cascades involving the production of type I interferon (IFN-I), serve as the primary bulwark against viral incursions. In the context of viral infections, the host cellular pattern recognition receptors (PRRs), encompassing Toll-like receptors (TLRs), retinoic acid-inducible gene I (RIG-I)-like receptors (RLRs), and DNA sensors, identify distinct pathogen-associated molecular patterns (PAMPs) to facilitate the synthesis of IFN-I (Wu and Chen, 2014; Liuyu et al., 2019). Empirical findings have elucidated that TBK1 plays a crucial role in the synthesis of IFN-I induced by diverse PRRs, including TLR3 (Kawai and Akira, 2010), RIG-I (Kato et al., 2006), melanoma differentiation-associated protein 5 (MDA5) (Belgnaoui et al., 2011), and cyclic GMP-AMP synthase (cGAS) (Zhang et al., 2019; Li et al., 2013; Cai et al., 2014). Consequently, an in-depth elucidation of the precise mechanisms underlying TBK1’s function within the IFN-I pathways, along with an exploration of their physiological consequences, could potentially reveal novel targets for the development of antiviral therapeutics (Akira et al., 2006; Chathuranga et al., 2021).

Post-translational modifications (PTMs) of TBK1 are highlighted by numerous studies as crucial for regulating its activity. Phosphorylation is a critical PTM of signaling molecules on tyrosine, serine, threonine, or histidine residues, thereby regulating downstream signaling networks (Leijten et al., 2022). Phosphorylation of TBK1 at Ser-172 is essential for its kinase activity, which is critical for TBK1-mediated IRF3 activation and IFN-β induction (McCoy et al., 2008; Lei et al., 2010). Xuelian Li et al. investigated that phosphorylation of TBK1 at Tyr179 plays an important role in regulating TBK1 activity by priming its autophosphorylation at Ser-172, (Li et al., 2017) which indicated that phosphorylation of TBK1 at Tyr may also be a crucial PTM for innate immunity. Src-related non-receptor tyrosine kinases (SFKs), which are classified into three subfamilies—Lyn-related (Lyn, Hck, Lck, Blk), Src-related (Src, Yes, Fyn, Fgr), and SFK-related (Brk, Frk, Srm)—phosphorylate diverse allosteric molecules, thereby regulating cell proliferation, migration, differentiation, and survival (Ingley, 2008; Kim et al., 2009). Previous studies have reported that Src activates TBK1 by phosphorylating its Tyr179 residue (Li et al., 2017). Conversely, Lck, Hck, and Fgr directly phosphorylate TBK1 at Tyr354/394, thus inhibiting its activation (Liu et al., 2017). Given the high homology among SFKs and their similar regulatory mechanisms, it is feasible that other SFKs may also play significant roles in the regulation of the TBK1-mediated IFN-I pathway.

Frk, a member of SFKs, acts as a key regulatory protein across various malignancies (Yim et al., 2009b). Initially identified as a tumor suppressor that halts cell proliferation and suppresses tumorigenesis in breast cancer, colon cancer, and glioma (Chandrasekharan et al., 2002; Yim et al., 2009b; Shi et al., 2015), numerous studies have demonstrated that Frk also contributes to the progression of hepatocellular carcinoma, pancreatic cancer, and leukemia (Hosoya et al., 2005; Chen et al., 2013; Je et al., 2014). Frk modulates various cellular processes via its kinase activity, including the inhibition of cyclin D1 nuclear accumulation, facilitation of N-cadherin/β-catenin complex assembly, regulation of JNK/c-Jun signaling, and prevention of PTEN ubiquitination and degradation (Yim et al., 2009b; Zhou et al., 2012; Hua et al., 2014; Yim et al., 2009a). Despite our previous findings suggesting that Frk may act as a key modulator in HIV infection (Wang et al., 2022), its role in modulating antiviral innate immunity is poorly understood.

In this study, we demonstrate that Frk acts as a positive regulator of antiviral immune responses in both *in vitro* and *in vivo* settings. Mechanistically, Frk directly interacts with TBK1, phosphorylating it at Tyr174 and Tyr179, thus promoting K63-linked ubiquitination of TBK1 and enabling the subsequent activation of IRF3, which is essential for the activation of IFN-I pathways.

## Materials and methods

### Ethics statement

All animal studies were approved by the Animal Experiment Administration Committee of Army Medical University and were conducted in accordance with governmental guidelines and institutional policies for the Care and Use of Laboratory Animals.

### Mice strains

Homozygous Frk^–/–^mice (from Cyagen Biosciences) were bred under specific pathogen-free conditions at the National Engineering Research Center of Immunological Products. Six-week-old male specific pathogen-free (SPF) Frk^–/–^ mice and their WT littermates used in the experiments were bred separately and euthanized by cervical vertebra dislocation.

### Reagents and plasmids

The following chemical reagents and antibodies were used in this study: anti-TBK1 (3504), anti-phospho-TBK1 (5483), anti-IRF3 (4302), anti-phospho-IRF3 (29047), rabbit anti-phospho-Tyr (8954), rabbit anti-Flag (14793), rabbit anti-HA (14031), mice anti-Myc (2276), fluorescent Alexa 488-conjugated anti-mice IgG (4408), fluorescent Alexa 594-conjugated anti-rabbit IgG (8889), and goat anti-rabbit IgG (5127), all from Cell Signaling Technology; rabbit anti-Frk (16197-1-AP) from Proteintech; anti-Flag M2 Affinity Gel (A2220) and rabbit anti-GAPDH (G9545), both from Sigma-Aldrich; Anti-HA Magnetic Beads from MCE (HY-K0201); rabbit anti-GST (CW0085M) from Cwbio; Protein G Sepharose 4 Fast Flow was purchased from GE Healthcare; DAPI (C1005), from Beyotime. Expression constructs for Flag-RIG-I, Flag-RIG-I-N, Flag-MAVS, Flag-TBK1, Flag-IRF3, Flag-cGAS, Flag-TRAF3, and HA-cGAS were obtained from Dr. B. Ge (Tongji University, Shanghai, China); Flag-STING was obtained from Dr. B. Sun (Shanghai Institute of Biochemistry and Cell Biology, Shanghai, China); cDNAs encoding Src, Frk, and Lyn were obtained from Dr. J. Han (Xiamen University, Fujian, China). HA-STING, HA-TBK1 and Myc-Ub K63 were stored in our labs. Site-directed point mutagenesis of TBK1 and Frk was performed using the QuickMutation™ Plus Mutagenesis Kit (D0208, Beyotime) according to the manufacturer’s instructions.

### Cells and viruses

Mice peritoneal macrophages, RAW264.7, HEK293T, and Vero cells were maintained in Dulbecco’s modified Eagle’s medium (SH30022.01, HyClone) supplemented with 10% (v/v) heat-inactivated fetal bovine serum (SH30406.05, HyClone) and 100 U/mL penicillin and streptomycin (SV30010, HyClone). HSV-1, and VSV were obtained from Dr. B. Ge (Tongji University, Shanghai, China).

### Isolation of mice peritoneal macrophages

To prepare mice peritoneal macrophages, 6-week-old homozygous Frk^–/–^ mice and their WT littermates were injected with 2 mL of 4% Brucella Broth. Three days later, peritoneal lavage fluid was collected from the mice, and the cells were washed three times with phosphate buffered saline (PBS). Peritoneal macrophages were cultured in DMEM supplemented with 10% FBS and 100 U/mL penicillin and streptomycin.

### Virus infection

VSV and HSV-1 were collected from the supernatants of infected Vero cells. For *in vitro* infection, RAW264.7, HEK293T, and peritoneal macrophages (2 × 10^6^ cells) were cultured in DMEM for 12 h and infected with VSV (MOI = 1) or HSV-1 (MOI = 5) for the indicated times. For *in vivo* infection, 6-week-old male homozygous Frk^–/–^ mice and their WT littermates were infected with VSV (5 × 10^7^ PFU per mouse) by intravenous injection for 24 h.

### Transfection and RNA knockdown by lentiviral vectors

HEK293T cells were transiently transfected with polyethylenimine (23966-2, Polysciences) according to the manufacturer’s protocol. RAW264.7 cells were transfected with INVI DNA RNA Transfection Reagent (IV1216025, Invigentech). For Frk knockdown, HEK293T cells were transfected with 10 μg pSPAX2, 5 μg pMD2.G, and 10 μg of shFrk or control vectors using Lipofiter™ (HB-TRLF, Hanbio). Supernatants contained virions were collected at 48 h and 72 h after transfection and virions were isolated by ultracentrifugation. The viral titer was determined via TCID50 assay in HEK293T cells by observing GFP fluorescence. Frk-specific shRNA (5′-GATCCGCGGCCAACATTTGAGACCCTGCATTTTCAAGAGA AATGCAGGGTCTCAAATGTTGGCCGTTTTTTG-3′) and control shRNA (5′- GATCCGTTCTCCGAACGTGTCACGTAAT TCAAGAGATTACGTGACACGTTCGGAGAATTTTTTC-3′) were used to knock down Frk in RAW264.7 cells.

### RNA interference

Small interfering RNAs (siRNAs) were transfected with jetPRIME (101000027, Polyplus). For Frk interference in RAW264.7 cells, the Frk-specific siRNA was purchased from Santacruz (sc-39232). For Frk interference in HEK293T cells, the target sequence for Frk employed in this study is as follows: 5′-GGAGUACCUAGAACCCUAUTT-3′.

### Quantitative real-time PCR

Cells were incubated for 12 h without serum and infected with viruses for the indicated time. Total RNA was extracted using RNAiso Plus (9109, Takara) according to the manufacturer’s instructions. RNA (1 μg) was reverse transcribed using a PrimeScript™ RT Reagent Kit (RR037, Takara) to generate cDNA. TB Green^®^ Premix Ex Taq™ II (RR820A, Takara) was utilized for quantitative real-time RT-PCR analysis. All values were normalized to the level of *GAPDH* mRNA, and relative expression was calculated using the comparative cycle threshold (2^–ΔΔCT^) method. Specific primers used for RT–PCR assays are listed in Supplementary Table 1.

### ELISA

Cells were incubated for 12 h without serum and infected with viruses for the indicated time. A Mice IFN-beta ELISA Kit (42400-1, R&D systems) was used to measure immunoreactive IFN-β in supernatants from RAW264.7 cells.

### Immunoprecipitation and western blotting

For immunoprecipitation, HEK293T cells were transfected with the indicated plasmids. After 48 h, the cells were washed three times with ice-cold PBS. The cells were lysed in lysis buffer (50 mM Tris pH 7.4, 150 mM NaCl, 1% Triton X-100, and 1 mM EDTA, pH8.0) supplemented with a protease inhibitor cocktail (04693159001, Roche), 1 mM PMSF, 1 mM Na3VO4, and 1 mM NaF. After 30 min on ice, the lysates were centrifuged for 15 min at 13,200 rpm and 4°C to remove debris. The cell lysates were incubated with anti-Flag M2 Affinity Gel or anti-HA Magnetic Beads at 4°C overnight. For immunoprecipitation of endogenous proteins, peritoneal macrophages or RAW264.7 cells were lysed, and the lysates were incubated with specific antibodies and Protein G Sepharose 4 Fast Flow at 4°C overnight. The Sepharose samples were centrifuged and washed three times with ice-cold PBST (1% Triton X-100 in PBS). For immunoblotting, the precipitates or cell lysates were boiled in 1× SDS loading buffer at 100°C for 10 min and then analyzed via immunoblot assay.

### GST pulldown

GST fusion proteins were expressed in *E. coli* BL-21(DE3) (CB105-01, Tiangen Biotech) according to the manufacturer’s instructions. The lysates from RAW264.7 cells were incubated with GST-fusion proteins conjugated to glutathione beads at 4°C for 4 h. After centrifugation and washing three times with ice-cold PBS, the beads were incubated in 1× SDS loading buffer at 100°C for 10 min and then analyzed via immunoblot assay.

### Dual-luciferase reporter assay

HEK293T cells were transiently transfected with IFN-β-Luc, pRL-TK, and other vectors as indicated for 24 h. A Dual-Luciferase Reporter Assay System (RG028, Beyotime) was used to detect luciferase activity according to the manufacturer’s instructions.

### Cell staining and confocal microscopy

HEK293T cells were transfected with the appropriate plasmids for 48 h and infected with HSV-1 or VSV for the indicated times. Peritoneal macrophages were infected with viruses directly. Cells were fixed with 4% formaldehyde for 20 min at room temperature, permeabilized for 30 min in PBS containing 0.3% Triton X-100, and then blocked for 1 h at 4°C in a blocking buffer (1% BSA in PBS). Subsequently, the cells were incubated with the indicated primary antibodies at 4°C overnight and secondary antibodies at room temperature for 1 h. After staining with DAPI, images were obtained using a Confocal Microscope ZEISS LSM 780.

### Post-translational modifications (PTMs) analysis with PTMcode 2

The phospho-tyrosine of TBK1 was analyzed using PTMcode 2 online tools at the following website: https://ptmcode.embl.de/index.cgi.

### Statistical analysis

Data are expressed as mean ± standard error of the mean (SEM). GraphPad Prism7 was used for statistical analysis. The statistical tests conducted in this study are indicated in the figure legends as follows: *P < 0.05, **P < 0.01, ***P < 0.001, ****P < 0.0001, two-tailed unpaired Student’s *t*-test. The sample sizes, reproducibility of experiments and the statistical tests used are presented in the figure legends. ZEN 2.1 on Zeiss LSM 780 confocal laser microscopy system was used for immunohistochemistry data collection and analysis. CFX Manager Software v3.0 on BioRad CFX96 Touch was used for qRT-PCR data collection and analysis. Bio-Rad ChemDoc Touch was used for western blot data collection and analysis.

## Results

### Frk positively regulates IFN-β signaling

**FIGURE 1:**
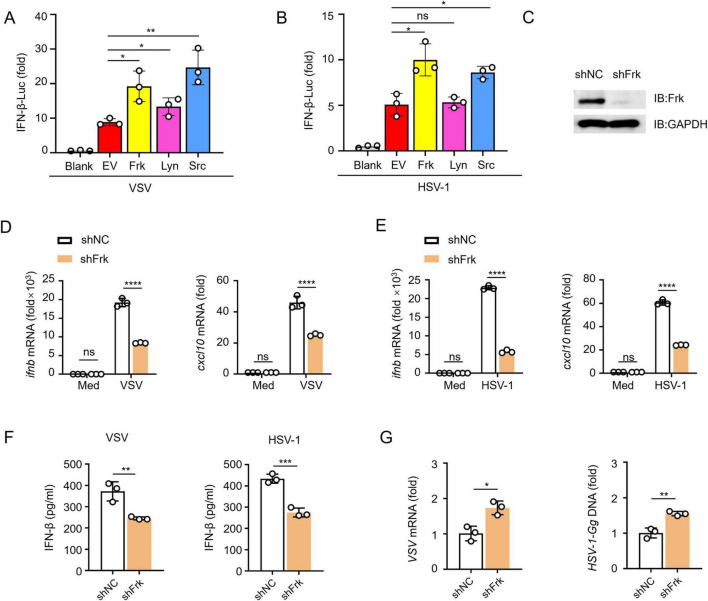
Frk knockdown impairs antiviral innate immunity. **(A,B)** Luciferase assay of IFN-β activity in HEK293T cells expressing various kinases (*n* = 3). **(C)** Impact of Frk shRNA in RAW264.7 cells. **(D,E)** *ifnb* and *cxcl10* mRNA levels in RAW264.7 cells transfected with Frk or control shRNA and infected with VSV **(A)** or HSV-1 **(B)** for 12 h (*n* = 3). **(F)** ELISA of IFN-β in the supernatants from **(D,E)** (*n* = 3). **(G)** Virus replication as in **(D,E)** for 12 h (*n* = 3). The data are representative of at least three independent experiments. The data are the means ± SEMs. *P < 0.5, **P < 0.01, ***P < 0.001, and ****P < 0.0001 (two-tailed unpaired Student’s *t*-test).

To investigate the function of Frk in the antiviral innate immune response, Frk, Lyn and Src were transfected into HEK293T cells and subjected to the infection with VSV and HSV-1, followed by a dual-luciferase assay. The results demonstrated a significant enhancement of the IFN-β luciferase signal upon overexpression of Frk and Src (Figures 1A, B). Src is known to facilitate IFN-I signaling (Li et al., 2017), suggesting that Frk may similarly operate within IFN-I pathways. Next, we analyzed Frk gene expression using the GEO database (Bajwa et al., 2016; Nissim et al., 2012), the analysis revealed diminished Frk mRNA levels in both hepatitis B virus (HBV)-associated acute liver failure patients and influenza-stimulated human plasmacytoid dendritic cells (Supplementary Figures 1A, B). These findings implied that Frk may play a role in the physiological and pathological processes associated with virus infection. Therefore, we transfected HEK239T cells with either a negative control siRNA or Frk-specific siRNA and observed a decrease in Frk expression following transfection with Frk-specific siRNA (Supplementary Figure 1C). As expected, we observed decreased mRNA levels of *IFNB* and *CXCL10* in Frk knockdown HEK293T cells compared to the control cells (Supplementary Figures 1D, E). Additionally, there were increased levels of VSV mRNA or HSV-1 genomic DNA in the knockdown cells (Supplementary Figure 1F). To further confirm the function of Frk, we achieved Frk knockdown in RAW264.7 cells via short hairpin (sh)RNA transfection (Figure 1C). Upon stimulation with VSV or HSV-1, significantly impaired mRNA expression of *ifnb* and *cxcl10* was observed in Frk-knockdown macrophages as compared to their control counterparts (Figures 1D, E). Consistently, Frk knockdown significantly reduced IFN-β production and increased the viral replication in RAW264.7 cells post-infection (Figures 1F, G). Taken together, these results suggested that Frk may play an important role in promoting IFN-β signaling in response to viral infection.

### Frk interacts with TBK1

**FIGURE 2:**
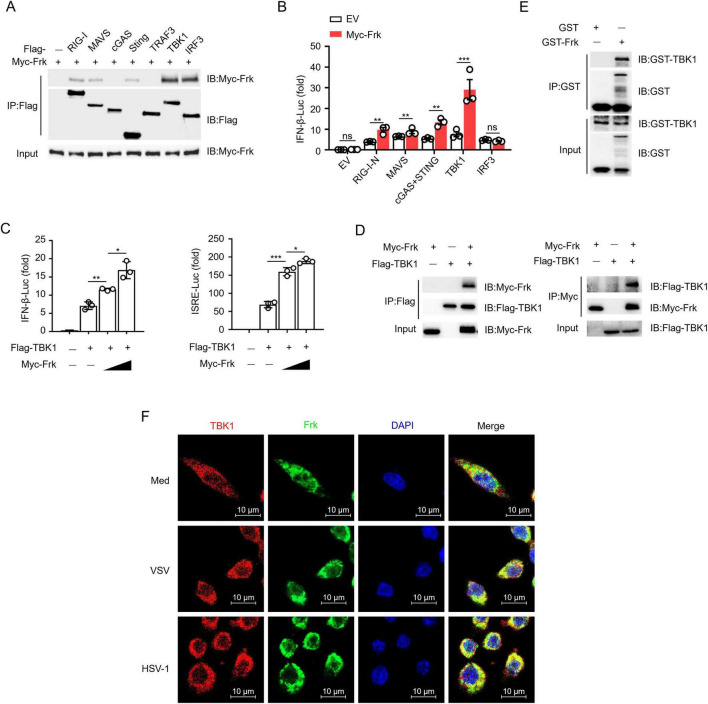
Frk directly interacts with TBK1. **(A)** Immunoassay of lysates from HEK293T cells expressing various vectors. **(B)** Luciferase assay of IFN-β activation in HEK293T cells expressing various vectors (*n* = 3). **(C)** Luciferase assay of IFN-β and ISRE activity in HEK293T cells expressing different concentrations of Frk (*n* = 3). **(D)** Immunoassay of lysates from HEK293T cells expressing various vectors. **(E)** Direct binding of GST-Frk to endogenous TBK1 from peritoneal macrophages. **(F)** Endogenous interaction of Frk and TBK1 in VSV or HSV infected RAW264.7 cells for indicated times. The data are representative of at least three independent experiments. The data are the means ± SEMs. *P < 0.05, **P < 0.01 and ***P < 0.001 (two-tailed unpaired Student’s *t*-test).

To identify the direct target molecule of Frk in IFN-β signaling, we conducted a coimmunoprecipitation screening experiment and found that Frk exhibited a high affinity for TBK1 and IRF3 (Figure 2A). Furthermore, dual-luciferase assay results indicated that Frk overexpression enhanced IFN-β luciferase activity induced by RIG-I-N, MAVS, STING, and TBK1, with no effect on IRF3 (Figure 2B). Subsequent experiments demonstrated that Frk dose-dependently enhanced IFN-β and Interferon Stimulated Response Element (ISRE) luciferase activity mediated by TBK1 (Figure 2C). These results suggested that Frk may target TBK1 to enhance IFN-β signaling. The interaction between Frk and TBK1 was further confirmed by both forward and reverse co-immunoprecipitation (Co-IP) experiments (Figure 2D). A GST pulldown assay revealed that Frk directly interacts with endogenous TBK1 in peritoneal macrophages (Figure 2E). Furthermore, immunofluorescence confocal microscopy demonstrated efficient colocalization of Frk with TBK1 post-viral infection (Figure 2F). These findings suggested that the activation of IFN-β signaling by Frk is likely through its interaction with TBK1.

### Frk enhanced TBK1-mediated IFN-β signaling through its kinase activity

**FIGURE 3:**
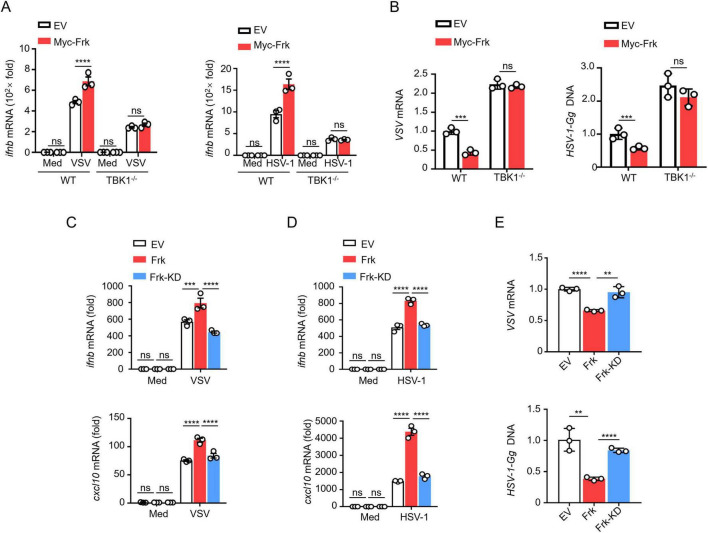
Frk promotes TBK1 activation dependent on its kinase activity. **(A)** *ifnb* mRNA levels in WT and TBK1^–/–^ RAW264.7 cells expressing various vectors and infected with VSV or HSV-1 for 12 h (*n* = 3). **(B)** Virus replication levels as in **(A)**. **(C,D)** *ifnb* and *cxcl10* mRNA levels in RAW264.7 cells expressing empty victor, WT or K262R Frk infected with VSV or HSV-1 (*n* = 3). **(E)** The virus replication levels as in **(C,D)**. The data are representative of at least three independent experiments. The data are the means ± SEMs. **P < 0.01, ***P < 0.001, and ****P < 0.0001 (two-tailed unpaired Student’s *t*-test).

Given that Frk interacts with TBK1, we examined whether Frk enhances IFN-β expression through TBK1 in virus-infected macrophages. WT and TBK1^–/–^ RAW264.7 cells were transfected with either a control vector or Frk. Frk overexpression in WT RAW264.7 cells significantly elevated the IFN-β mRNA expression levels and inhibited the replication of VSV and HSV-1, otherwise in TBK1^–/–^ RAW264.7 cells (Figures 3A, B), suggesting that Frk primarily targets TBK1 to modulate the IFN-β signaling pathway.

Considering Frk is a protein tyrosine kinase, we assessed the role of its kinase activity in TBK1-mediated signaling. Frk becomes kinase-deficient when the lysine at position 262 in its ATP binding site is mutated to arginine (hereafter referred to as Frk-KD) (Jin and Craven, 2014). Thus, we transfected RAW264.7 and HEK293T cells with either Frk or Frk-KD, followed by infection with VSV or HSV-1, respectively. The results indicated that Frk-KD did not significantly increase the mRNA expression of *ifnb/IFNB* and *cxcl10/CXCL10* (Figures 3C, D and Supplementary Figures 2A, B). Furthermore, we found that viral replication was suppressed in cells overexpressing Frk, whereas no such inhibition was seen in cells overexpressing Frk-KD (Figure 3E and Supplementary Figure 2C). These findings suggested that Frk’s promotion of TBK1-mediated IFN-β signaling is dependent on its kinase activity.

### TBK1 Y174/179 phosphorylation is critical for Frk-mediated antiviral immunity

**FIGURE 4:**
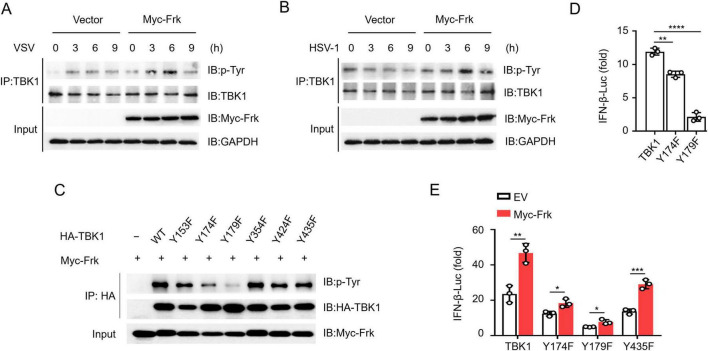
Frk phosphorylates TBK1 at Tyr174 and Tyr179. **(A,B)** Immunoassay of lysates from RAW264.7 cells which were transfected with Frk, or control vector infected with VSV or HSV-1. **(C)** Immunoassay of lysates from HEK293T cells expressing various vectors. **(D,E)** Luciferase assay of IFN-β activation in HEK293T cells expressing various vectors (*n* = 3). The data are the means ± SEMs. *P < 0.05, **P < 0.01, ***P < 0.001, and ****P < 0.0001 (two-tailed unpaired Student’s *t*-test).

To ascertain whether Frk phosphorylates TBK1 during infection and to elucidate the mechanism, we infected RAW264.7 cells with VSV and HSV-1. The results showed that Frk overexpression significantly increased tyrosine phosphorylation of TBK1 (Figures 4A, B). To pinpoint the specific tyrosine residues phosphorylated by Frk, we analyzed the potential tyrosine phosphorylation sites of TBK1 by using PTMcode 2: https://ptmcode.embl.de/index.cgi. Afterward, we generated TBK1 mutants with six conserved tyrosine residues replaced by phenylalanine (Y → F) (Supplementary Table 2) (Minguez et al., 2013). Immunoprecipitation following co-transfection of these TBK1 mutants with Frk in HEK293T cells revealed that TBK1(Y174F) and TBK1(Y179F) displayed significantly lower phospho-tyrosine levels (Figure 4C) and indicating that both Y174 and Y179 could be phosphorylation sites of Frk. Moreover, TBK1-mediated IFN-β luciferase activity was diminished following substitution with either TBK1(Y174F) or TBK1(Y179F) (Figure 4D). However, Frk was still able to enhance IFN-β luciferase activity mediated by TBK1(Y174F) or TBK1(Y179F), suggesting that the phosphorylation of Y174 and Y179 both play crucial roles in Frk-mediated IFN-I pathway (Figure 4E).

**FIGURE 5:**
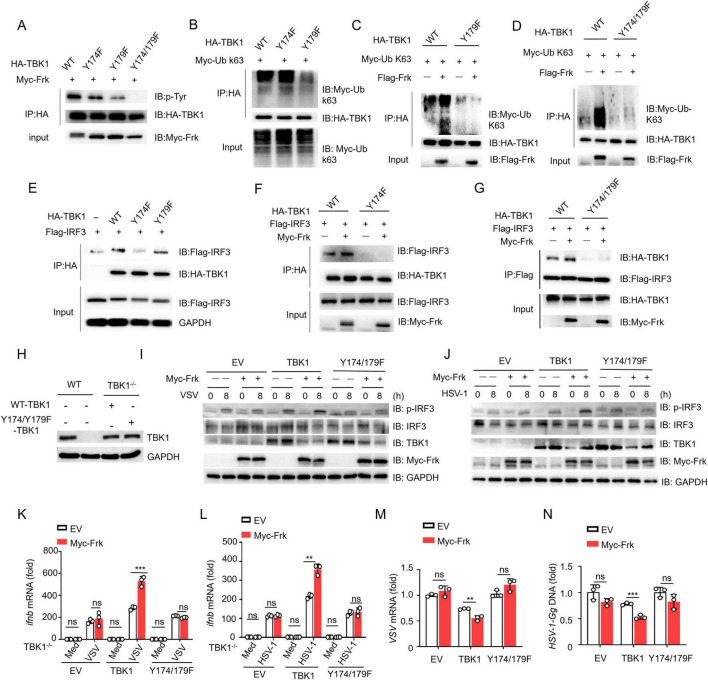
TBK1 Y174/179 phosphorylation is critical for Frk-mediated antiviral immunity. **(A-G)** Immunoassay of lysates from HEK293T cells expressing various vectors. **(H)** Expression of endogenous TBK1 in WT and TBK1^–/–^ RAW264.7 cells and re-introduced WT or Y174/Y179F TBK1 in TBK1^–/–^ RAW264.7 cells. **(I,J)** Immunoblot of lysates from TBK1^–/–^ RAW264.7 cells transfected with various vectors and infected with VSV or HSV-1 for indicated times. **(K,L)** *ifnb* mRNA levels in TBK1^–/–^ RAW264.7 cells transfected with various vectors and infected with VSV or HSV-1 for 12 h (*n* = 3). **(M,N)** Virus replication levels as in **(K,L)**. The data are representative of at least three independent experiments. The data are the means ± SEMs. **P < 0.01, and ***P < 0.001 (two-tailed unpaired Student’s *t*-test).

Next, we created a dual mutant, TBK1(Y174/179F), which showed no detectable tyrosine phosphorylation when catalyzed by Frk (Figure 5A). Therefore, we further investigated the regulation of Frk-mediated Type I interferon pathway by the two sites, respectively. Previous reports have demonstrated that TBK1 recruits and phosphorylates IRF3 to activate the IFN-I pathway (Hosoya et al., 2005), and K63-linked ubiquitination of TBK1 also facilitates this process (Song et al., 2016). We found that the phosphorylation of Y179 on TBK1 promoted its K63-linked ubiquitination (Figures 5B–D), while the phosphorylation of Y174 on TBK1 mediated the recruitment of IRF3 to TBK1 (Figures 5E–G). Xuelian Li et al. determined that phosphorylation of TBK1 at Y179 is important for autophosphorylation of TBK1 at Ser - 172, which is required for TBK1 activation (Li et al., 2017). Consistent with this, we found that the phosphorylation of tyrosine on TBK1 mediated by Frk also mediates the phosphorylation of TBK1 at Ser-172 (Supplementary Figure 3). Subsequently, we complemented TBK1 and TBK1(Y174/179F) into TBK1^–/–^ RAW264.7 cells in the presence of Frk. Western Blot results showed that the expression of the transfected TBK1 and TBK1(Y174/Y179F) was successful (Figure 5H). Following virus infection, we observed the restoration of IRF3 phosphorylation and IFNB mRNA expression in cells complemented with TBK1, with further enhancement upon Frk overexpression. Conversely, cells complemented with TBK1(Y174/179F) did not demonstrate a comparable rescue effect (Figures 5I–L). Consistently, Frk improved the efficiency of viral replication restriction via TBK1, with no such effect seen with TBK1(Y174/179F) (Figures 5M, N). Collectively, these data indicated that Frk facilitates TBK1-mediated IFN-β signaling through the phosphorylation of Tyr174 and Tyr179 on TBK1.

### Frk positively regulates antiviral immune response both *in vitro* and *in vivo*

**FIGURE 6:**
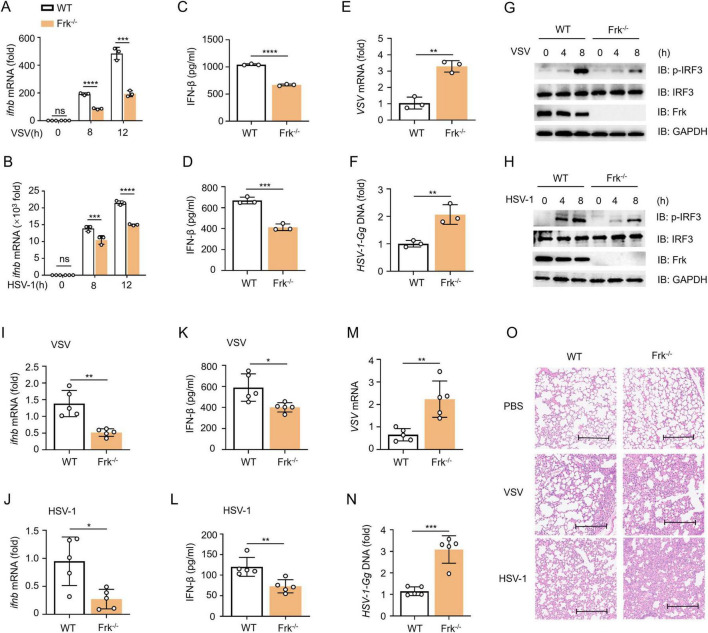
Frk positively regulates antiviral immune response both *in vitro* and *in vivo*. **(A,B)** *ifnb* mRNA levels in WT and Frk^–/–^ mice peritoneal macrophages infected with VSV **(A)** or HSV-1 **(B)** for indicated times (*n* = 3). **(C,D)** ELISA of IFN-β in the supernatants at 12 h from **(A,B)** (*n* = 3). **(E,F)** The virus replication levels as in **(A,B)** for 12 h (*n* = 3). **(G,H)** Immunoblot of lysates from WT and Frk^–/–^ mice peritoneal macrophages infected with VSV **(G)** or HSV-1 **(H)** for indicated times. **(I,J)** *ifnb* mRNA levels in lung of WT and Frk^–/–^ mice (*n* = 5 mice per group) infected for 24 h by intravenous injection of VSV or HSV-1(5 × 10^7^ PFU per mouse). **(K,L)** ELISA of IFN-β as in **(I,J)**. **(M,N)** The virus replication levels as in **(I,J)**. **(O)** Hematoxylin and eosin staining of lung sections from mice as in **(I,J)**. Scale bar: 260 μm. The data are representative of at least three independent experiments. The data are the means ± SEMs. *P < 0.05, **P < 0.01, ***P < 0.001, and ****P < 0.0001 (two-tailed unpaired Student’s *t*-test).

To further elucidate the role of Frk in IFN-β production, peritoneal macrophages isolated from wild-type (WT) and Frk knockout (Frk^–/–^) mice were infected with VSV or HSV-1. The results showed that virus-induced IFN-β production was substantially diminished, and viral replication was markedly enhanced in Frk^–/–^ macrophages (Figures 6A–F). Likewise, phosphorylation of IRF3 was also compromised in Frk^–/–^ macrophages (Figures 6G, H). Subsequently, the *in vivo* antiviral efficacy of Frk was examined using an intravenous viral injection model. In alignment with the *in vitro* findings, IFN-β secretion in the lungs was significantly diminished in Frk^–/–^ mice relative to wild-type mice following VSV or HSV-1 infection, concurrent with elevated viral replication (Figures 6I–N). In addition, Frk^–/–^ mice exhibited more severe lung injuries (Figure 6O). These *in vitro* and *in vivo* data indicated that Frk is an important positive regulator for antiviral immune responses.

## Discussion

TBK1 not only serves as a pivotal regulator in the synthesis of IFN-I but also contributes to the modulation of diverse signaling pathways, such as NF-κB signaling, autophagy, cell cycle progression, Ras-driven oncogenesis, and AKT pro-survival signaling, through its distinct PTMs (Alam et al., 2021). Therefore, therapeutic agents directly targeting TBK1 may elicit a range of unpredictable physiological responses and are not considered an ideal approach for stimulating antiviral immunity. Targeting specific regulators involved in modulating TBK1 PTMs could emerge as a potential therapeutic strategy for the management and prophylaxis of viral infections (Zhao and Zhao, 2019).

In this study, we identified Frk as a non-receptor tyrosine kinase capable of activating IFN-β expression. Inhibition of Frk expression diminishes the production of IFN-β triggered by VSV or HSV-1. This phenomenon accounts for the finding from the GEO database analysis that successful infection of hosts by HBV and influenza viruses correlates with reduced Frk expression. Consequently, modulating Frk activation could enhance therapeutic outcomes and the prognostic outlook for patients suffering from VSV or HSV-1 infections.

Our research provides significant insights into the mechanisms by which Frk modulates the host responses in antiviral innate immunity. Firstly, we found that Frk directly interacts with TBK1 to facilitate the activation of the IFN-β signaling pathway after VSV and HSV-1 infections. Moreover, as a member of SFKs, Frk phosphorylates TBK1 at residues Y174 and Y179. This process promotes K63-linked ubiquitination of TBK1, concurrent with the recruitment and activation of IRF3. Lastly, Frk positively regulates the antiviral immune responses both *in vitro* and *in vivo* settings. Our findings suggest that the activation of TBK1, mediated by Frk-dependent tyrosine kinase activity, is crucial for enhancing host antiviral defense during viral infections. However, prior studies have shown that numerous phosphokinases are involved in regulating TBK1 activity. For instance, Src can also phosphorylate Tyr179 of TBK1 (Li et al., 2017). We further explored the relationship between Src, Frk, and TBK1 and found that Frk mediates TBK1 activation independently of Src (Supplementary Figures 4, 5). Additionally, GSK3β, PPM1A, and PPM1B modulate TBK1 activity by altering the phosphorylation state of Ser172 in TBK1 (Lei et al., 2010; Xiang et al., 2016; Zhao et al., 2012). Protein tyrosine kinase 2 beta has been demonstrated to directly phosphorylate TBK1 at residue Y591, thereby enhancing TBK1 activation through increased oligomerization (Lin et al., 2023). In contrast, Shengduo Liu et al. found that certain SFK members, such as Lck/Hck/Fgr, directly phosphorylate TBK1 predominantly at residues Tyr394/354, leading to the inhibition of TBK1 activation (Liu et al., 2017). Therefore, further research is necessary to determine the specific agonists or inhibitors that target Frk to modulate the antiviral responses mediated by TBK1. Our team is committed to continuing our research in this direction.

K63-linked ubiquitination, in addition to phosphorylation, is a crucial post-translational modification for TBK1 activation (Huang et al., 2023; Liang et al., 2024; Li et al., 2019; Meng et al., 2021). Our study revealed that Frk phosphorylates TBK1, thereby promoting its K63-linked ubiquitination and the recruitment of IRF3. However, the mechanism by which Frk’s tyrosine phosphorylation of TBK1 drives its K63-linked ubiquitination remains elusive. Previous studies identified RING finger protein 128 (RNF128) as a pivotal E3 ubiquitin ligase that not only promotes K63-linked ubiquitination but also activation of TBK1 (Song et al., 2016). Furthermore, the viral E2 ubiquitin-conjugating enzyme pI215L, derived from African swine fever virus, was observed to strengthen the interaction between RNF138 and RNF128, thereby facilitating the degradation of RNF128. This led to a decrease in K63-linked polyubiquitination of TBK1 and a subsequent reduction in type I IFN production (Huang et al., 2021). Additionally, Dan Li et al. demonstrated that TBK1 acts as an E3 ubiquitin ligase capable of self-ubiquitylation in vitro, in the presence of the E2 enzyme UbcH5c, as well as *in vivo* (Li et al., 2019). Therefore, the mechanisms underlying the regulation of TBK1’s K63-linked ubiquitination by Frk-induced tyrosine phosphorylation will be another focus of our future research.

In conclusion, this study provides novel insights into the regulation of TBK1 activation and the TBK1-IRF3 signaling pathway through tyrosine phosphorylation. Given the crucial role of IFN-β in restricting viral infections, our findings may offer valuable therapeutic implications for drug development targeting virus-induced diseases.

## Data Availability

The raw data supporting the conclusions of this article will be made available by the authors, without undue reservation.
