# Supplementary material for: Frk positively regulates innate antiviral immunity by phosphorylating TBK1
Source: Front Microbiol. 2025 Feb 12;16:1525648. doi: 10.3389/fmicb.2025.1525648 (PMC11861356; doi:10.3389/fmicb.2025.1525648)
Supplement: Supplementary file 1 [file Data_Sheet_1.docx]

**Supporting information**

**
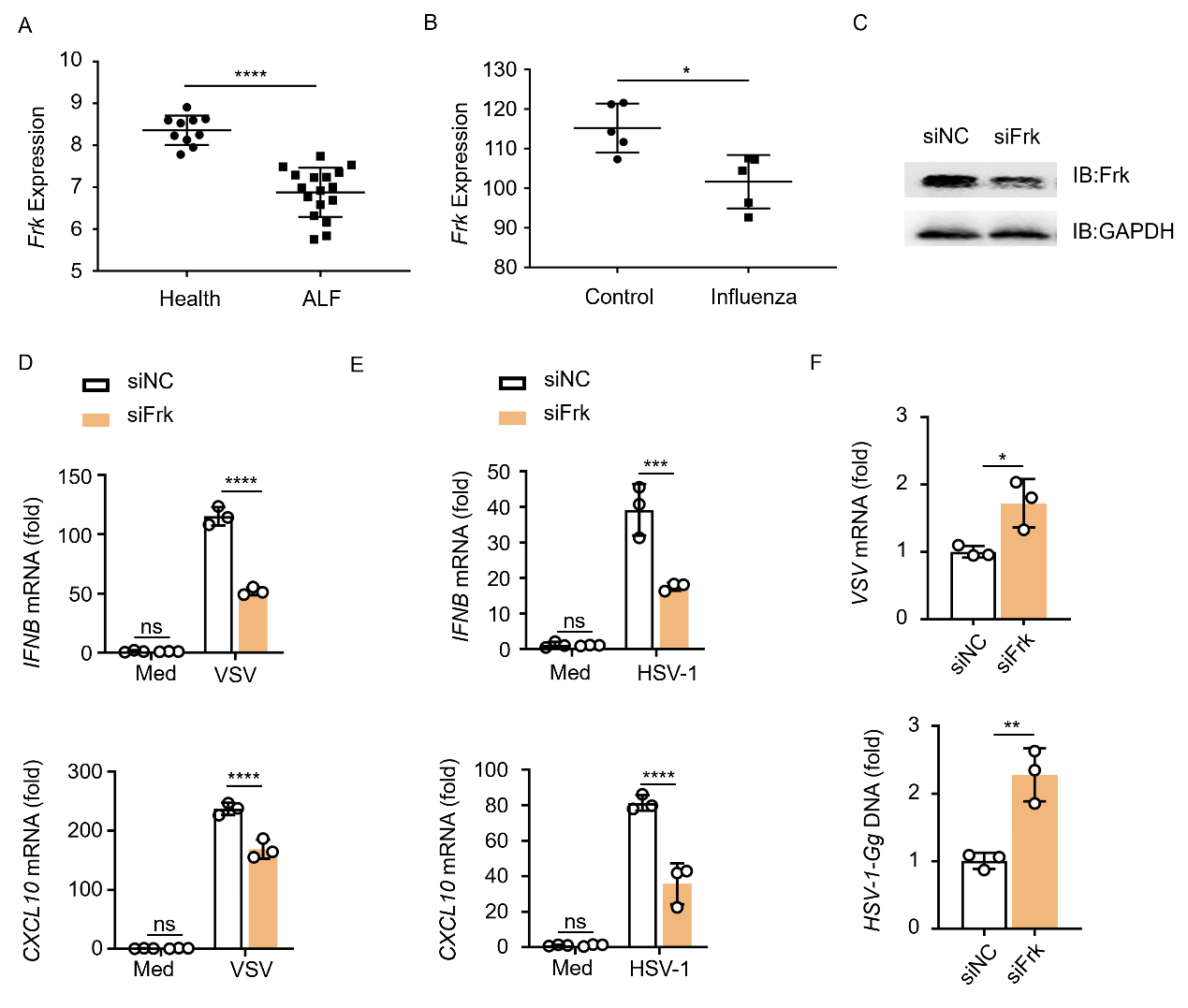
**

**Fig S1. Frk function in antiviral innate immunity.** (A and B) Frk expression analysis from GEO database. (C) Impact of Frk siRNA in HEK293T cells. (D and E) *IFNB* and *CXCL10* mRNA levels in HEK293T cells transfected with Frk or control siRNA and infected with VSV or HSV-1 for 12 h (n = 3). (F) The virus replication as in (D and E) for 12 h (n = 3). Data are representative of at least three independent experiments. Data are means ± SEMs. **P* < 0.5, ***P* < 0.01, ****P* < 0.001, and *****P* < 0.0001 (two-tailed unpaired Student's *t*-test).

**
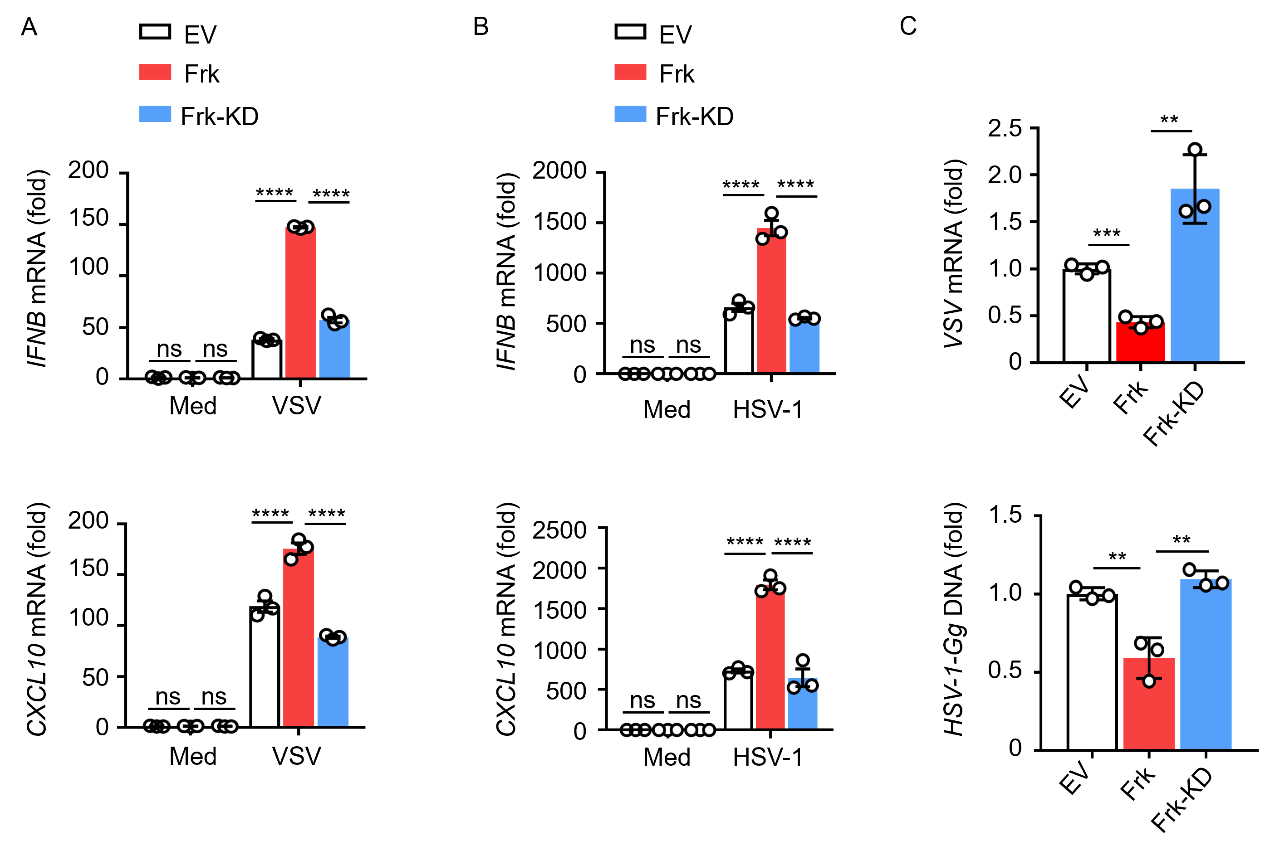
**

**Fig S2. Frk promotes TBK1 activation dependent on its kinase activity.** (A and B) *IFNB* and *CXCL10* mRNA levels in HEK293T cells expressing various vectors infected with VSV or HSV-1 (n = 3). (C) The virus replication levels as in (B and C). The data are representative of at least three independent experiments. The data are the means ± SEMs. ***P* < 0.01, ****P* < 0.001, and *****P* < 0.0001 (two-tailed unpaired Student’s *t*-test).

**
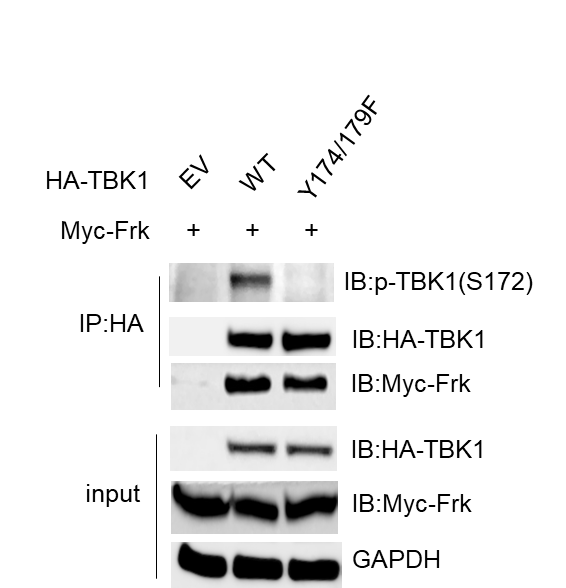
**

**Fig S3. Frk phosphorylates TBK1 at Tyr174 and Tyr179.** Immunoassay of lysates from HEK293T cells expressing various vectors. Data are representative of at least three independent experiments.


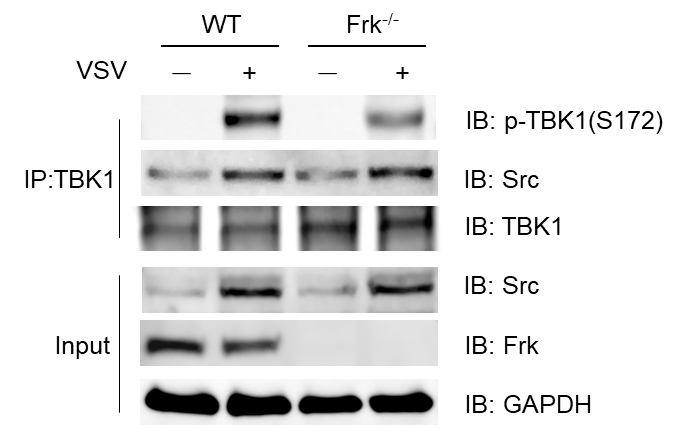


**Fig S4.** **Frk does not influence the expression of Src and its interaction with TBK1 but affects the phosphorylation of TBK1 at Ser172.** Immunoassay of lysates from WT and Frk^-/-^ RAW264.7 cells which were infected with VSV. Data are representative of at least three independent experiments.


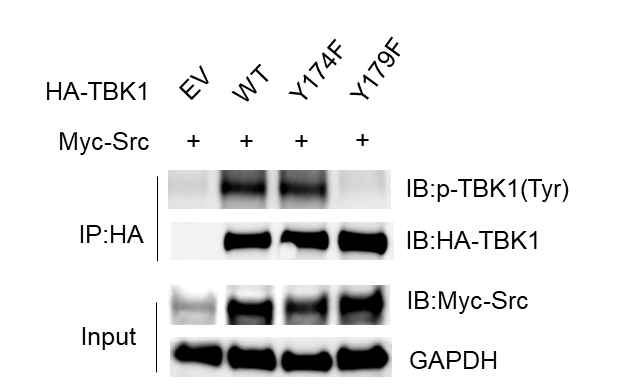


**Fig S5. Src specifically catalyzes the phosphorylation of Y179 without affecting the phosphorylation of Y174.** Immunoassay of lysates from HEK293T cells expressing various vectors. Data are representative of at least three independent experiments.

**Table S1.** **Primer sequences that are used to amplify human genes and mouse genes in real-time quantitative PCR.**

| Human *IFNB* | forward | TCTGGCACAACAGGTAGTAGGC |
| --- | --- | --- |
|  | reverse | GAGAAGCACAACAGGAG |
| Human *CXCL10* | forward | GGAACCTCCAGTCTCAGCACCA |
|  | reverse | AGACATCTCTTCTCACCCTTC |
|  | reverse | AGGAAGGCCTAAGGTCCACT |
| Human *GAPDH* | forward | GCAAATTCCATGGCACCGT |
|  | reverse | GCCCCACTTGATTTTGGAGG |
| Mouse *ifnb* | forward | AGTTACACTGCCTTTGCC |
|  | reverse | GTTGAGGACATCTCCCAC |
| Mouse *cxcl10* | forward | CCAAGTGCTGCCGTCATTTT |
|  | reverse | GATAGGCTCGCAGGGATGAT |
| Mouse *gapdh* | forward | CCCACTAACATCAAATGGGG |
|  | reverse | CCTTCCACAATGCCAAAGTT |
| VSV mRNA | forward | ACGGCGTACTTCCAGATGG |
|  | reverse | CTCGGTTCAAGATCCAGGT |
| *HSV-1-g* DNA | forward | TGGGACACATGCCTTCTTGG |
|  | reverse | ACCCTTAGTCAGACTCTGTTACTTACCC |

**Table S2. Potential tyrosine phosphorylation sites of TBK1 analyzed by** **PTMcode 2.**

| **PTM1** | **AA** | **POSITION** | **CONSERVATION** |
| --- | --- | --- | --- |
| phosphorylation | Y | 179 | 100 |
| phosphorylation | Y | 354 | 97 |
| phosphorylation | Y | 435 | 93 |
| phosphorylation | Y | 174 | 93 |
| phosphorylation | Y | 153 | 91 |
| phosphorylation | Y | 424 | 82 |
